# Psychological Benefits of a Sport-Based Program for Female Cancer Survivors: The Role of Social Connections

**DOI:** 10.3389/fpsyg.2021.751077

**Published:** 2021-11-26

**Authors:** Ilaria Durosini, Stefano Triberti, Valeria Sebri, Alice Viola Giudice, Paolo Guiddi, Gabriella Pravettoni

**Affiliations:** ^1^Applied Research Division for Cognitive and Psychological Science, IEO, European Institute of Oncology IRCCS, Milan, Italy; ^2^Department of Oncology and Hemato-Oncology, University of Milan, Milan, Italy

**Keywords:** cancer survivors (CSs), sport, quality of life, group cohesion, cancer

## Abstract

In the context of sports-based interventions for improving health and quality of life in chronic patients, participants could develop meaningful social relationships that affect their well-being as much as intervention activities. In this study, 80 female cancer survivors participated in a running-based group intervention (2 sessions/week; 1.5 h), while 51 acted as controls. The intervention lasted approximately 5 months. Unfortunately, the length of the intervention was reduced and sport activities were altered by the COVID-19 pandemic and lockdown mid-intervention, while the shared therapy sessions continued online. This possibly altered the results, as anxiety, depression, and physical aspects did not show significant differences between the experimental and control groups after the intervention. Participants reported positive comments on the experience as a whole, especially regarding the positive influence of the newly developed social connections. This was corroborated by significant correlations between group perceived cohesion and anxiety, depression, self-efficacy, and positive experience with the group psychological support. Overall, it is possible to suggest that in the program an important role was played by social connections and support, peer influence and the group experience, preserving positive experiential qualities of the intervention even if it was altered by external circumstances.

## Introduction

The advances in cancer treatment lead to a reduction in the number of deaths related to this disease. Despite the improvement in survival rate, the experience of cancer leads people to physical and psychological consequences that persist over time (Sterba et al., 2014). Even after treatments, survivors have to cope with adverse effects (e.g., vascular complications, mood disorders, and cognitive dysfunctions) that affect their quality of life and general well-being (Ahmad et al., 2015; Williams and Jeanetta, 2016; Marzorati et al., 2017). Cancer-related illness could be experienced as a trauma associated with high negative emotions and impairments in different areas of present life and future planning.

Previous studies have indicated that sports performance improves quality of life and physical functioning (Northey et al., 2019), controlling for example body weight by decreasing fat (Cheema and Gaul, 2006). Furthermore, sports help patients to improve psychological functioning and cognitive skills (such as attention, memory, and decision-making processes; Pesce et al., 2013; Zimmer et al., 2016), and strengthen the overall sense of identity and self-esteem. Identity can also be enhanced thanks to an educational approach through (and not only *of*) the physical on both individual and group support levels (Liao and Masters, 2016). Firstly, cancer patients develop notable introspection processes through the discovery of their own abilities and strengths and weaknesses, a knowledge particularly important to build a new sense of Self after the illness experience (Hodge and Lonsdale, 2011). Such abilities can be adapted and transferred in other life domains to increase personal skills, for example in cancer management (Pierce et al., 2018; Sebri et al., 2020). According to reviews and meta-analyses in the field, sport-based interventions generally have positive effects on breast cancer survivors’ quality of life and health management (Craft et al., 2012; Bluethmann et al., 2015; Neil-Sztramko et al., 2019), although some specific physical activities appear less effective than others (e.g., TaiChi; Yan et al., 2014). Physical activity and sports were reported in the literature as associated with passion and positive emotions in breast cancer survivor (Burke et al., 2012); qualitative studies on the experience of breast cancer survivors within quality of life interventions focused on sports help to understand the meaningful psychological outcomes of these activities, ranging from feeling positively challenged, sense of closure, growth and personal strength, and also improving one’s own body image and finding new social support resources (Burke and Sabiston, 2010; Brunet et al., 2013).

However, the effectiveness of sports programs largely depends on the motivation and adherence of participants. Recent studies highlighted that motivation can change over time and peer/group support is relevant to dedicate time and enjoy activities by having other people involved in the same physical activities with similar efforts and emotional difficulties (Elmagd et al., 2018; Durosini et al., 2021a; Savioni et al., 2021). Indeed, social support promotes patients’ commitment and engagement in activities that demand lifestyle changes, such as sport (Richardson et al., 2017; Savioni et al., 2021). The perception of being part of a group fosters individual motivation thanks to the creation of new social bonds (Cho et al., 2020; Durosini et al., 2021a) that promote deeper reflection on one’s own motivation by impacting the general experience of a group as well as one’s own personal objectives (Novick et al., 2011). In the context of sports-based interventions for improving health and quality of life in chronic patients, participants could develop meaningful social relationships that affect their well-being as much as intervention activities.

The involvement of adaptive cognitive skills and social support perception is particularly useful over the current COVID-19 pandemic and its related mental health issues (Marazziti et al., 2020), particularly worrying for people with cancer experience (Renzi et al., 2019; Brivio et al., 2021a). Wang et al. (2020) evidenced that cancer patients tend to show a higher level of anxiety, distress, and depression compared to the healthy population, as shown in Italy by Brivio et al. (2021b). Additionally, COVID-19 has caused inconveniences for people who need regular assessment at hospitals, for example, due to lack of treatment (Millar et al., 2020; Swainston et al., 2020), which can increase cancer patients’ worry and distress.

The overall aim of this study was to evaluate the benefits related to a running group-based program with female cancer survivors. Specifically, the main goal was to evaluate the changes in physical and mental well-being following participation in a group running program, group therapy sessions and receiving nutritional counseling. It was expected that the sport-based program would lead to significant improvements in psychological and physical well-being compared to the participants included in the control group. However, the intervention was partially altered by the COVID-19 pandemic and lockdown, which made it impossible for the participants to complete all the running activities that had been originally planned, as the beginning of the project was delayed according to lockdown national regulations (Durosini et al., 2021b). Secondarily, the running activities were not performed within closed spaces in the end but in the open and abiding by safety rules (the participants wore masks and respected social distancing). Thirdly, while the main running activities were completed, a marathon that was originally set as the final “goal” for the participants, and held important motivational value, was canceled. The participants had been still able to attend all the focused formation and therapy sessions online.

## Methods

### Participants

This study involved a total of 131 women. All participants lived in Italy and received a diagnosis of cancer in the past. The overall mean age of the sample was 50.1 + 6.8 years. Inclusion criteria were: female participants; with a history of cancer; no evidence of disease at the time of the intervention; age >18 years; ability to read and understand Italian; agreement to participate in the study.

Eighty women voluntarily took part in a sports group intervention program to promote quality of life that combined physical exercises (running activity) with group psychological sessions with psychologists (*M*_*age*_ = 48.70, *SD*_*age*_ = 6.25; age range: 31–60), the Pink is Good project (see section “Intervention”). 33% of the women originally involved in the Pink is Good project refused to participate or dropped out, mostly because of cancer recurrence, difficulty in adhering to the intervention activities, or the delay related to the COVID-19 lockdown.

The remaining 51 women were included in the control group and continued their life normally, without participating in the program (*M*_*age*_ = 52.26, *SD*_*age*_ = 6.99; age range: 41–67).

No significant differences were found in the type of cancer [*X^2^* = 2.953 (4), *p* = 0.566] and in level of education [*X*^2^ = 0.384 (2), *p* = 0.825] between the sport-based intervention group and the controls (see Table 1 for sample information). However, it should be noted that a significant difference was found between the two groups regarding age, *t*(128) = −3.02, *p* < 0.01.

**TABLE 1:** Characteristics of participants included in the experimental and control groups.

	**Experimental group (*N* = 80)**		**Control group (*N* = 51)**			
	***n***	***%***	***n***	***%***	***X*^2^ (df)**	***p***
Type of cancer					2.953 (4)	0.566
Breast	65	91.5	44	93.6		
Ovarian	4	5.6	2	4.3		
Uterus	1	1.4	0	0		
Leukemia	0	0	1	2.1		
Melanoma	1	1.4	0	0		
Level of education					0.384 (2)	0.825
Secondary school	3	3.8	3	6.0		
High school	34	42.5	20	40.0		
University degree or advanced training course	43	53.8	27	54.0		

All participants performed the running activities and attended the support groups.

### Procedure

All the participants were informed about the aim of the research and provided informed consent before participating in the study. Participants included in the sports group voluntarily took part in the Pink is Good project promoted by Fondazione Umberto Veronesi (Italy), while controls did not. Data were collected at the beginning (June 2020) and at the end (October 2020) of the intervention in each group.

Ethical approval was granted by the European Institute of Oncology, IRCCS (n. R1248/20-IEO1313).

#### Intervention

Women included in the intervention group voluntarily took part in a program that combined physical exercises with psychological support, the “Pink is Good” project. Despite the COVID-19 pandemic, the main activities and contents have been successfully adapted allowing the project to be carried out safely for all participants. For a period of at least 5 months, reduced compared to the original version, women participated in a running group training program with a professional trainer and received advice from a nutritionist regarding their eating habits. All the trainers have extensive professional experience in fitness and organized personalized training programs specifically designed to meet the abilities of each cancer survivor. During the intervention, women also received group psychological support from psychologists, focused on their personal goals within the intervention (see Table 2 and Supplementary Table 1 for more details on the intervention program).

**TABLE 2:** Intervention program.

	**Methodology**	**Aim**
*Physical training*	*Group:* Small groups (4–15 participants) *Approach:* Personalized training for each participant *Session:* Two sessions per week, 1.5 h/each	Running techniques are used to train participants to run long distances (ranging from 5 to 21 km) Sessions aim for example to improve muscle strength, flexibility, muscle resistance, and cardio-pulmonary function
*Nutrition training*	*Group:* Small groups (4–15 participants) *Approach:* Active involvement of participants *Session:* Three sessions, 2 h/each	*Session 1:* - Educate on tertiary prevention and nutrition management for running - Provide practical information and ideas: how to go shopping, how to easily prepare healthy dishes *Session 2:* - Support change in eating habits: practical advice *Session 3:* - Encourage to improve the care in the quality and type of food, achieving greater food awareness
*Psychological support*	*Group:* Small groups (4–15 participants) *Approach:* Collaborative intervention. Each participant was actively involved in a psychological session *Session:* Three sessions, 2 h/each	*Session 1:* - Help each group to express the expectations of belonging to the “Pink is Good” team - Give to the group a space for discussion on their experience - Define the emerging criticalities - Work on group cohesion, resources and the hardships of being a working group with a common goal *Session 2:* - Work on the efforts and resources of being a “Pink is Good” team *Session 3:* - Individual and social evaluation of the experience

Participants included in the control group continued their usual daily life without involvement in any health intervention.

### Measures

All the participants were asked to complete the following questionnaires at the beginning (T1) and at the end (T2) of the intervention.

#### Patient Health Questionnaire-9

The PHQ-9 (Kroenke et al., 2001; Mazzotti et al., 2003; Picardi et al., 2005) is a self-report questionnaire that assesses clinical depression in adults. Its statement scores each of the 9 DSM-IV criteria ranging from “0” (not at all) to “3” (nearly every day). Kroenke et al. (2001) recommended a cut-off point of ≥10 to assess depression. In this study, the scale revealed a good reliability equal to 0.819 at T1 and 0.862 at T2.

#### State Trait Anxiety Inventory

The STAI (Spielberger, 1970; Spielberger et al., 1983; Pedrabissi and Santinello, 1989) is a self-report questionnaire designed to measure the vulnerability to anxiety experiences. It explores anxiety both as an emotional state and as a stable personality trait through a 4-point Likert scale. For the purpose of this study, we used only the state anxiety subscale. Research has shown that the STAI has adequate concurrent validity and internal consistency (Spielberger, 2013). In the present study, the scale showed a good Cronbach’s alpha equal to 0.954 at T1 and 0.955 at T2.

#### General Perceived Self-Efficacy

The GSE (Schwarzer and Jerusalem, 1995; Sholz et al., 2002) is a self-report scale that provides a global reference for participants’ coping ability in a wide range of demanding situations. It consists of 10 items with responses based on a 4-point Likert-type scale ranging from 1 (Not at all true) to 4 (Completely true). In this study, the scale revealed an optimal reliability equal to 0.903 at T1 and 0.908 at T2.

#### Physical Aspects

In order to assess physical changes in participants included in the experimental and control group, we assessed the Body Mass Index (BMI) and the Waist to Hip Ratio (WHR) of women.

Secondarily, the subsequent measures were administered to the experimental group only, because they regarded evaluation or experience of the specific experimental activities.

#### Self-Assessment Manikin

Self-Assessment Manikin (SAM) (Bradley and Lang, 1994) is a set of three single-item scales. Participants are asked to indicate in a continuum of five human-like figures (manikins) their own emotions on a variety of dimensions: valence (sadness–happiness), arousal (tenseness–calmness), and dominance (mastery). Participants were invited to indicate their own emotions (a) before (b) during, and (c) after the physical training session. Data was collected two times: at the beginning of the training program (T1) and at the end of the training program (T2).

#### Group Environment Questionnaire

The GEQ (Carron et al., 1985; Andreaggi et al., 2000) is a self-report scale that assesses the cohesion in sport teams through four factors. The first dimension, *Individual Attractions to the Group–Social* (ATG-S), refers to the team member’s acceptance within the group and impressions of social interactions. The second dimension, *Individual Attractions to the Group–Task* (ATG-T), refers to a group member’s feelings about personal involvement in relation to shared group productivity and goals. The third factor, *Group Integration– Social* (GI-S), refers to a team member’s sense of group similarity, closeness, and bonding as a social unit. Lastly, the fourth factor, *Group Integration–Task* (GI-T), refers to an individual’s beliefs about team similarity, closeness, and bonding around the group’s task. Data collected in T2 in the experimental group highlighted a Cronbach’s alpha equal to 0.899.

#### Experience of Psychological Support

This is an *ad hoc* questionnaire that assesses participants’ experience of group psychological sessions with clinical psychologists received during the project through 16 items. The scale was rated on a 5-point Likert scale. Higher scores suggested a greater experience with the mental health professionals. The scale was created based on the Parent Experience of Assessment Scale (PEAS; Austin et al., 2018) and included items such as “*I felt that my opinion was valued; The psychologist was genuinely interested in helping us; I felt the psychologist respected me; I learned a tremendous amount about myself from this psychological sessions.*” The scale showed a good Cronbach’s alpha equal to 0.884.

### Data Analysis

First of all, to test for differences between the experimental and the control group, mixed design analysis of variance (ANOVA) with the data collected at the beginning (T1) and at the end (T2) of the intervention both in the experimental and control groups was performed on depression, anxiety, self-efficacy and physical aspects.

On the data collected in the experimental group only, we conducted repeated measures analysis of variance (ANOVA) on the perceived valence, arousal, and dominance of emotions (SEM).

Finally, to test the relationship between the cohesion perceived by the women within the experimental group and psychological aspects, we carried out Pearson’s correlation analyses between the cohesion in sport teams (GEQ), negative emotions (depression and anxiety), and general self-efficacy. Additionally, we explored the relationship between team cohesion and the experience related to the psychological support received by the experimental group within the intervention.

All data were analyzed using the Statistical Package for the Social Sciences (SPSS) *Version 21*. A power analysis was conducted to ensure meaningful and statistically significant results (Cohen, 1988). Analyses were run with the software G^∗^Power (Faul et al., 2007), with power (1 – b) set at 0.80, a medium effect size (one-tailed test), a 5% level of significance. Data highlighted that we needed a total sample size of 34 participants in order to detect a medium effect size. Our sample estimates were very close to our hypothetical suggestions, ensuring that there were adequate levels of power for the detection of effect. A *p*-value of <0.05 was considered as statistically significant.

## Results

Consistently with the data emerging from the global literature on the psychological impact due to the COVID-19 pandemic (e.g., Sebri et al., 2021), participants included in the experimental and in the control groups showed an increased level of anxiety and negative emotions over time (Table 3).

**TABLE 3:** Descriptive statistics and ANOVA results.

	**Intervention group**		**Control group**		***F***	***p***	**η*2***
	***Pre***	***Post***	***Pre***	***Post***			
PHQ-9	4.19 (2.53)	5.32 (4.60)	8.00 (4.89)	8.39 (4.39)	1.231	0.270	0.010
STAI-S	36.24 (10.27)	39.17 (12.52)	42.56 (12.45)	44.30 (12.65)	0.380	0.539	0.003
GSE	29.41 (4.55)	29.54 (5.31)	28.51 (4.93)	27.93 (4.84)	0.940	0.334	0.008
BMI	22.82 (3.60)	22.24 (2.98)	23.69 (3.86)	23.74 (3.51)	2.669	0.105	0.026
WHR	0.85 (0.18)	0.79 (0.15)	0.84 (0.39)	0.88 (0.41)	3.534	0.064	0.047

*PHQ-9, Patient Health Questionnaire-9; STAI-S, State and Trait Anxiety Inventory-State; GSE, general perceived self-efficacy; BMI, Body Mass Index; WHR, Waist to Hip Ratio.*

Regarding physical changes, results revealed that physical training programs led to changes in the body. We observed that participants who attended the Pink is Good project highlighted a reduction in the BMI, yet this index remained in the normal range.

Additionally, even if not statistically significant, participants included in the experimental group highlighted a reduction of the WHR, although not significant (Table 3).

Analyses conducted on the experimental group only revealed that participants experienced more pleasant and intense emotions after the training session. Specifically, data highlighted significant differences in the pleasantness of emotions (i.e., valence) and the level of arousal during the session. In T1, participants experienced more pleasant emotional state [*F*(2) = 11.49, *p* < 0.001, η^2^ = 0.141] at the end of the session (*M* = 8.51, *SD* = 0.83) than at the beginning of the session (*M* = 7.76, *SD* = 1.54). Significant differences in the pleasantness of emotions experienced at the beginning and at the end of the training session emerged also in T2. Indeed, after 5 months from the start of the project, participants tend to perceive increased emotional pleasantness [*F*(2) = 26.78, *p* < 0.001; η^2^ = 0.256] during the running session (beginning: *M* = 6.90, *SD* = 2.12; end: *M* = 8.38, *SD* = 1.30).

Similar results emerged for the intensity of emotions (i.e., arousal). Participants experienced more intense emotions at the end of the session (T1 – *M* = 7.79, *SD* = 1.66; T2 – *M* = 7.47; *SD* = 1.87) than to the beginning of the session (T1 – *M* = 6.90, *SD* = 1.81; T2 – *M* = 6.58, *SD* = 1.98). Statistical significant differences emerged both in T1 [*F*(2) = 14.50, *p* < 0.001; η^2^ = 0.172] [*F*(2) = 12.45, *p* < 0.001; η^2^ = 0.138] and T2. The dominance of emotions did not yield significant results.

Pearson’s correlations highlighted a significant negative relationship between group cohesion in sport teams and depression. The impressions of social interactions and personal acceptance within the group (ATG-S) and the sense of group similarity, closeness, and bonding as a social unit (GI-S) are negatively related with the symptoms associated with clinical depression in women who participated in the intervention group (ATG-S: *r* = −0.264, *p* = 0.019; GI-S: *r* = −0.247, *p* = 0.028). In the same line, the personal acceptance within the group and impressions of social interactions is negatively related with anxiety (ATG-S: *r* = −0.240, *p* = 0.034). The participants’ impressions of social interactions and personal acceptance within the sport group (ATG-S) and their feelings about personal involvement in relation to productivity and shared group goals (ATG-T) are significantly related with the general self-efficacy (ATG-S: *r* = 0.303, *p* < 0.01; ATG-T: *r* = 0.265, *p* = 0.018). Additionally, all the components included in the cohesion in sport teams (both in task and social aspects) are related to the positive experience in group psychological support sessions (ATG-S: *r* = 0.540, *p* < 0.001; ATG-T: *r* = 0.531, *p* < 0.001; GI-T: *r* = 0.636, *p* < 0.001; GI-S: *r* = 0.556, *p* < 0.001; see Table 4).

**TABLE 4:** Correlations between GEQ and psychological variables.

	**PHQ-9**	**STAI-S**	**GSE**	**Experience of psychological support**
GEQ_ATG-S	−0.264*	−0.240*	0.303**	0.540**
GEQ_ATG-T	−0.200	−0.129	0.265*	0.531**
GEQ_GI-T	−0.218	−0.180	0.195	0.636**
GEQ_GI-S	−0.247*	−0.218	0.121	0.556**

*p < 0.05, **p < 0.01.

*GEQ_ATG-S, Individual Attractions to the Group–Social subscale; GEQ_ATG-T, Individual Attractions to the Group–Task subscale; GEQ_GI-T, Group Integration–Task subscale; GEQ_GI-S, Group Integration–Social subscale.*

## Discussion

This research analyzed a sport-based program to improve the quality of life in breast cancer survivors. Unfortunately, the 2020 edition of the “Pink is Good” program was affected by the COVID-19 pandemic which partially hindered the planned activities so that the researchers had to adapt the program to the repeated lockdown periods and the necessity to adhere to the national safety recommendations when the participants were expected to run. The paucity of significant differences between the groups, interpreted here primarily in terms of the impossibility for the participants to enjoy in full the sport activities of the intervention, is consistent with the literature showing that physically active people were more exposed to well-being and emotional issues during the lockdowns than others (Zhang et al., 2020; Szcześniak et al., 2021), and some felt even stigmatized as some narratives in the media depicted physically active people as those more likely to ignore stay-at-home orders and to put others’ health at risk (Ranieri, 2020).

Regarding comparison measurements, results did not show significant differences between the experimental and the control group in terms of anxiety, depression, self-efficacy, and physical aspects. Nevertheless, the opportunities for social confrontation within shared psychological support sessions were positively associated with improvements.

Specifically, participants in the experimental group improved their emotions during the running activities. Despite this, the general level of anxiety and negative emotions improved slightly for participants. This may be related to the COVID-19 pandemic that forced people to change their life habits and to perform physical activities with some limitations. Consistently with the data emerging from the global literature, the COVID-19 pandemic had a relevant impact on psychological well-being (Durosini et al., 2021b).

It is possible to suggest that in the “Pink is Good” program an important role was played by social connections and support, peer influence and the group experience, as corroborated by qualitative research on the same project that highlighted how the social components of the program played a fundamental motivational role to participants’ engagement (Durosini et al., 2021a). Despite the limitations, the participants were able to interact and share their experiences with cancer, survivorship and everyday life, and often they reported (as brief comments on the intervention experience) the social and friendship opportunities as the main factor among their take-home benefits. This is corroborated by correlations featuring a measure for perceived quality of the group, which show a clear pattern, with group quality appearing inversely related to anxiety and depression, and positively related to generalized self-efficacy and an adapted measure of satisfaction with the group therapy activities.

As the main limitation of the study, the COVID-19 pandemic and related safety regulations certainly affected the implementation of the intervention (e.g., altering the way and the frequency the sport activities were conducted), so that results should be considered only partially indicative of the original hypotheses and research design. Yet, this study represented the opportunity to explore which factors within a psychological intervention may be positive and useful for cancer survivors when an intervention is altered by external circumstances. Relevant indexes of participants’ quality of life (depression and anxiety) or participants’ commitment to healthcare management and the intervention itself (self-efficacy and satisfaction with group therapy) appeared related to the perceived quality of the peer group, created by the participants and enriched during the intervention months. It is possible that both sport activities and group cohesion would obtain significant effects within an intervention more extended in time, similar to how “Pink is Good” was originally conceived before the COVID-19 related alterations, as previous research reports that peer relationships and the building of novel social support resources can promote participants’ motivation and health outcomes (Queen et al., 2017; Chen and Zhao, 2020; Durosini et al., 2021a). This is consistent with research showing that support groups may be more welcomed and beneficial to breast cancer patients if proposed and described in innovative ways (Green et al., 2018), for example as sport groups that will share self-empowering activities. As a limitation of the present study, participants’ motivation was not analyzed as a possible influence on final outcomes. Participants’ motivations may be addressed in future research aimed at exploring the overall influencing factors on the intervention. Also, future studies could assess the long term effects of different cancer types on the study outcomes.

It should be noted that the results pertaining to improvements in emotions and the role of peer support can be connected to the experimental group only, as these factors were measured only in the participants who engaged in the group/running activities. Future research on analogous interventions may add broader comparison measurements of emotional state and social support that would assess the control group as well, in order to demonstrate that these improvements were indeed related to the experience of the intervention.

Another limitation of the present study is that it was evaluated as a whole so that it is not possible to clarify the extent to which any psychological benefits were due to running or psychological support or both (e.g., Mustian et al., 2017). Future studies on similar composite interventions may feature systematic analysis of participants’ testimony to account for their experience with more specificity. Future research should emphasize and analyze more systematically the social/group factor within psychological interventions for quality of life in chronic patients, in that it may be a fundamental factor for health outcomes and recovery which holds still even in the face of external circumstances and alterations. Finally, while we hypothesized that the COVID-19 pandemic and related regulations may have affected the results by restricting the program activities, at the time of data collection it was not possible to add additional measures to account for the pandemic’s perceived impact among research participants and so to explore its effect on results as a covariate. It may be important, for analogous psychological interventions conducted in the years of COVID-19, to take this aspect into consideration in order to account for the effects of the pandemic on participants’ responses to intervention activities.

## Data Availability Statement

The raw data supporting the conclusions of this article will be made available by the authors, without undue reservation.

## Ethics Statement

The studies involving human participants were reviewed and approved by the European Institute of Oncology, IRCCS. The patients/participants provided their written informed consent to participate in this study.

## Author Contributions

ID conceptualized the study presented in the manuscript, collected the data, and wrote the first draft. ST and VS contributed to the conceptualization and writing. ID, ST, and VS ran the analyses. ST edited the manuscript. AG and PG helped the data collection and edited the manuscript. GP contributed to the important intellectual content and supervised the whole process. All authors contributed to the article and approved the submitted version.

## Conflict of Interest

The authors declare that the research was conducted in the absence of any commercial or financial relationships that could be construed as a potential conflict of interest.

## Publisher’s Note

All claims expressed in this article are solely those of the authors and do not necessarily represent those of their affiliated organizations, or those of the publisher, the editors and the reviewers. Any product that may be evaluated in this article, or claim that may be made by its manufacturer, is not guaranteed or endorsed by the publisher.
