# Supplementary material for: Psychological Benefits of a Sport-Based Program for Female Cancer Survivors: The Role of Social Connections
Source: Front Psychol. 2021 Nov 26;12:751077. doi: 10.3389/fpsyg.2021.751077 (PMC8664561; doi:10.3389/fpsyg.2021.751077)
Supplement: Supplementary file 1 [file Table_1.docx]

| **FREQUENCY** | **INTENSITY** | **TYPE** | **TIME** | **VOLUME** | **PROGRESSION** | **GOALS** |
| --- | --- | --- | --- | --- | --- | --- |
| 2 days/week | 40-59% | Aerobic exercises | > 20min < + di 1h | Approx. 400 meet min/week | Start slow and go slow | ↑ Cardio pulmonary function  ↑ Glucose tolerance  ↑ HDL  ↓ LDL  ↓ Fat Mass  ↓ Fatigue |
| 1day/week | 1 to 3 sets per muscle group/10-12 reps in sets or circuit training | Strength exercises | It depends from the number of exercises | >500 < 1000 meet min/week | Gradual progression | ↑ Muscle Mass  ↑ Muscle Strength  ↑ Power  ↑ Muscle Resistance  ↑ Bone muscle density  ↑ Performance  ↓ Fatigue  ↓ Fat Mass |
| 2days/week | 40-59% | Flexibility exercises | 10-30sec for each exercises | Approx. 400 meet min/week | Gradual progression | ↑ ROM (amplitude of body movement) |
| 2days/week | 40-59% | Coordination/running technique exercises | > 15 < 20mins for each training |  | Gradual progression | ↑ Neural function and cognitive processes  ↑ Efficiency of the technical gesture  ↑ Running economy |

*Adapted from: American College of Sports Medicine*
